# Exploring Misconceptions of Palliative Care Among Patients With Hepatocellular Carcinoma: A Pilot Study

**DOI:** 10.1177/10499091241268423

**Published:** 2024-08-19

**Authors:** Mostafa Abasseri, Shakira Hoque, Kim Caldwell, Linda Sheahan, Slavica Kochovska, Meera Agar, Amany Zekry

**Affiliations:** 1 School of Medicine and Health, 7800 UNSW, Sydney, NSW, Australia; 2 Gastroenterology and Hepatology Department, St George Hospital, Sydney, NSW, Australia; 3 Palliative Medicine, Calvary Hospital Kogarah, Kogarah, NSW, Australia; 4 Clinical Ethics Service, South Eastern Sydney Local Health District, Randwick, NSW, Australia; 5 Sydney Health Ethics, 4334 The University of Sydney, Camperdown, NSW, Australia; 6 St George and Sutherland Clinical Campus, 7800 UNSW Medicine & Health, Sydney, NSW, Australia; 7 Palliative Medicine Department, St George Hospital, Sydney, NSW, Australia; 8 Faculty of Science, Medicine and Health, 90119 University of Wollongong, Wollongong, NSW, Australia; 9 IMPACCT, Faculty of Health, 1994 University of Technology, Sydney, NSW, Australia

**Keywords:** palliative care, hepatocellular carcinoma, patients, attitudes, communication barriers

## Abstract

**Background:**

Hepatocellular carcinoma is a burdensome form of liver cancer with an increasing global prevalence. Emerging evidence has shown that early palliative care introduction at diagnosis of any life-limiting illness improves patient and carer outcomes. Despite this, patients with hepatocellular carcinoma usually receive palliative care late. These patients are important stakeholders in the provision of palliative care, but their perceived barriers regarding its delivery are poorly defined.

**Aim:**

This **pilot** study aimed to identify the barriers perceived by patients to integrating palliative care into the hepatocellular carcinoma treatment algorithm.

**Design:**

Patients living with hepatocellular carcinoma undertook semi-structured interviews about their perceptions of palliative care. We compared these perceptions before and after providing a brief explanation of palliative care. Interview data was inductively coded in NVivo 12 (2018) and thematically analysed.

**Results:**

Twenty-one patients were interviewed. 16 perceived palliative care to mean end-of-life therapy, and nine participants had no prior knowledge of palliative care. After hearing a definition of palliative care, 17 participants reported changed positive attitudes. Seven participants supported a name change, including four participants who continued to reject palliative care following the explanation due to the negative stigma associated with the term ‘palliative care’.

**Conclusion:**

There is significant misperception about the purpose of palliative care among patients with hepatocellular carcinoma, constituting a barrier to early integration. This can be feasibly addressed with a two-folded educational and renaming initiative to dispel patient misconceptions regarding palliative care. Effective strategies to achieve this should be developed and tested with relevant stakeholders, particularly patients.

## Introduction

Palliative care is a medical approach that improves the quality-of-life of patients with life-limiting illnesses, along with providing support for their families and caregivers. This is achieved through proactive identification, assessment and treatment of the physical, psychosocial and spiritual aspects of health.^1,2^

Importantly, there has been an increase in the early uptake of palliative care in the treatment algorithm for a variety of life-limiting conditions.^3,4^ Extensive evidence supports the implementation of palliative care at the time of diagnosis in these diseases, with early specialist palliative care associated with statistically significant improvements in quality-of-life, symptom relief and survival.^5-9^ Despite this, the implementation of palliative care in hepatocellular carcinoma at the time of diagnosis remains suboptimal, with patients receiving palliative care only at the terminal stage of their disease.^3^

Hepatocellular carcinoma is the most common primary liver malignancy and the second-leading cause of cancer-related mortality globally.^10^ It presents unique treatment and prognostic challenges due to its unpredictable progression^11^ secondary to its association with end-stage liver disease.^10^ Despite its escalating incidence, the implementation of palliative care at the time of hepatocellular carcinoma diagnosis is often deferred until the terminal stage, hindering the potential benefits associated with early palliative care input.^12^ This delay is exacerbated by the interplay between tumour-related factors and hepatic impairment in end-stage liver disease, increasing complexity in both treatment decisions and disease trajectory.^13^ These factors also contribute significantly to emotional distress, positioning hepatocellular carcinoma among the cancers with the highest psychological burden.^14^

Several factors hindering palliative care uptake in hepatocellular carcinoma have been previously reported.^15^ These entail limitations in current data,^16-19^ unpredictable disease trajectory delaying the initiation of palliative care discussions,^20,21^ stigma among clinicians reducing palliative care referrals,^22-24^ and a significant gap in understanding patient-perceived barriers to the initiation of early palliative care. This final point informs the objective of this study: to identify patient-perceived barriers to integrating palliative care into the hepatocellular carcinoma treatment algorithm.

## Methodology

### Design

We conducted a qualitative **pilot** study in the form of semi-structured interviews with patients living with hepatocellular carcinoma. This study followed the COnsolidated criteria for REporting Qualitative research (COREQ) guidelines.^25^ Our study design utilised an interpretive framework, acknowledging the subjective and context-dependent nature of our participants’ perspectives.^26^ This approach aligned with our study aim, as we sought to understand these individuals’ lived experiences.

### Setting & Ethics

This project was conducted at the St. George Hospital, Sydney, from April 2022 to August 2022. Ethics approval for the study was granted by the South Eastern Sydney Local Health District Human Research Ethics Committee (no. 2021/ETH11094).

### Participants and Recruitment

Patients diagnosed with any stage of hepatocellular carcinoma were invited to participate in the study during their clinic visits. Purposive sampling was used to balance migrant and non-migrant patients to reflect the culturally and linguistically diverse nature of the hepatocellular carcinoma population and to facilitate data saturation. Potential participants received information and consent forms and a study withdrawal form. Eligible participants were adult patients (≥18 years) with a diagnosis of hepatocellular carcinoma and an ability to provide written informed consent (supported by an interpreter for those from non-English speaking backgrounds). Participants were excluded if they had cognitive impairment due to severe hepatic encephalopathy that would preclude their ability to provide written informed consent. Recruitment ceased as data redundancy was achieved, indicating thematic saturation.^27^

### Data Collection

A topic guide for the semi-structured interviews was developed from published works in palliative care and hepatology^28-30^ to explore patients’ perceptions of palliative care and their hospital care needs (Table 1). Questions were designed to be broad, including both open- and closed-ended questions, and participants were encouraged to lead interviews with minimal facilitator input to minimise emotional distress. Participants were asked to define their opinions and emotions towards palliative care before and after a standardised definition of palliative care was provided (Table 2).

**Table 1.** Topic Guide and Interview Questions.

Topic	Questions Asked
Perceptions of palliative care	• When you hear the term “palliative care”, what is the first thing that comes to mind for you?
	• Would you accept palliative care as part of your treatment?
	• How would you describe palliative care in your own words?
	• [Following brief description of palliative care] does this change how you feel about palliative care?
	• In your opinion, when would be the best time for patients with liver cancer to learn about palliative care?
Needs of patients in the context of hospital care	• What has your experience been like since being diagnosed with liver cancer?
	• What sources of strength do you look to when life is difficult?
	• Would you find it helpful to talk to someone who could help you explore the issues of spirituality and faith?
	• What is/are your most burdensome symptoms?
	• Are you happy with your current care?
	• How has the illness affected your everyday life?
	• Can you tell me about some of the concerns or worries that you or your family have had since your diagnosis?

These questions were used as prompts, and the subsequent responses were categorised into groups according to similar themes.

**Table 2.** Standardised Definition of Palliative Care Provided to Participants.

Palliative care is specialised medical care for people facing a serious illness that focuses on providing patients with relief from symptoms and stress of a serious illness with the goal of improving quality of life for the patient and the family.
Palliative care is appropriate for patients at any age and at any stage in a serious illness and can be provided along with the recommended standard of care treatment for your liver cancer.
In studies comparing palliative care to routine oncological care, participants who received palliative care as an adjunct to active therapy experienced improved quality of life, survival, and decreased symptom burden.^a^

Definition adapted from.^28^

^a^These studies are randomised controlled trials with cohorts consisting of patients living with advanced cancer.^5-9^

Interviews were held face-to-face in a private room in the clinic. These were conducted and audio-recorded by one researcher (MA) who did not have a close relationship with participants. Participants were accompanied by caregivers per their request. Interpreters were used for participants not proficient in English. Interviews were recorded and transcribed, with written informed consent from participants. These ranged from 6 to 47 minutes in length. Interviews were not repeated, and transcripts were not returned for participant review. No field notes were taken.

### Data Analysis

The coding team (MA, SH) inductively consensus-coded transcribed interview data using NVivo 12 software (2018). Vaismoradi et al.’s thematic content analysis method was used to derive themes, a descriptive qualitative approach utilised to systematically code large amounts of textual information to determine patterns between themes.^27^ Codes were organised into themes and subthemes, and interview transcripts were reviewed multiple times throughout this process to ensure that the themes reflected original data. Participant demographics were extracted from electronic medical records and reported descriptively to understand the study sample. These were described using Microsoft Excel (2016). This paper identifies participant quotes by participant role (P = patient), followed by an assigned numerical code corresponding to their chronological study number.

## Results

Of the 24 patients invited to partake in interviews, three declined, and none withdrew consent, resulting in a final sample of 21 participants. All patients who declined cited discomfort discussing palliative care. Participants’ demographic and clinical data are depicted in Table 3.

**Table 3.** Participant Characteristics.

Participant Characteristics	Variable	Number	Percentage (%)
Age	Median 63 years; Range 49 – 92 years		
Sex	Male	17	81
	Female	4	19
Country of origin	Australia	9	43
	United Kingdom	2	9
	China	5	23
	Bangladesh	1	5
	Thailand	1	5
	Lebanon	1	5
	Syria	1	5
	Vietnam	1	5
Religion	No religion	11	53
	Christianity	4	19
	Islam	3	14
	Buddhism	3	14
Aetiology of HCC	Chronic hepatitis B	7	33
	Chronic hepatitis C	9	43
	NASH	2	10
	Alcohol	3	14
BCLC-stage	A B C D	10	48
		4	19
		6	29
		1	4
Days since diagnosis of HCC	Median 371 days; Range 7 – 1095 days		
History of clinical features	Pain	18	86
	Ascites	9	43
	Hepatic Encephalopathy^a^	3	14
	Portal Hypertension	13	62
	Varices	9	43
	Spontaneous Bacterial Peritonitis	1	5
Underlying cirrhosis	Yes	15	71
	No	6	29

HCC, Hepatocellular Carcinoma; NASH, Non-alcoholic steatohepatitis; BCLC, Barcelona Clinic Liver Cancer staging system (BCLC-A representing early disease, and BCLC-D correlating to advanced disease).

^a^Note that cases of hepatic encephalopathy were historical and participants were cognisant at the time of interviews, and thus deemed capable of providing informed consent.

**Figure 1.**
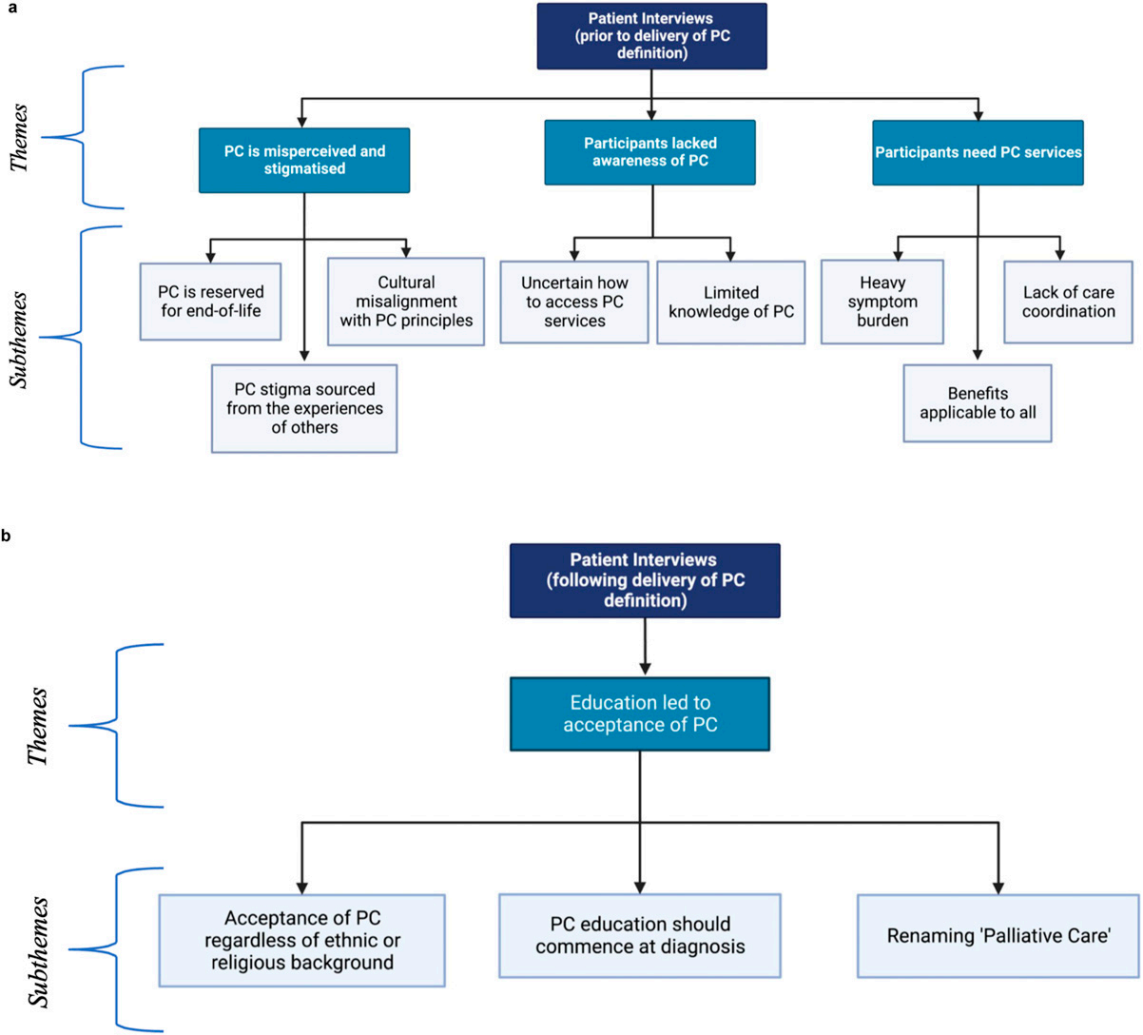
(A) Thematic illustration of themes and subthemes arising from participant interviews prior to brief definition of palliative care’s role in hepatocellular carcinoma. Created with BioRender.com. (B) Thematic illustration of themes and subthemes arising from participant interviews following brief definition of palliative care’s role in hepatocellular carcinoma. Created with BioRender.com.

Four key themes were identified through thematic content analysis (Figure 1(A), and 1(B)). These were (1) Palliative care is commonly misperceived and stigmatised, (2) Participants lacked awareness of palliative care, (3) Participants need palliative care services, and (4) Education led to acceptance of palliative care. Each of these themes had several subthemes, reported below. The coding table is presented in Supplementary Material 1.

### Theme 1: Palliative Care is Misperceived and Stigmatised

#### Palliative Care is Reserved for the End-Of-Life

A misunderstanding that palliative care was reserved for the end-of-life and represented halting active treatment was common among participants (n = 16), who expressed concern that accepting palliative care would mean “giving up” on their battle with cancer. Often, this translated to a stark rejection of palliative care, with three participants stating that they would prefer voluntary-assisted dying over palliative care. P4 emphasised, *“I’m very pleased that the voluntary euthanasia thing is probably going to pass. I would rather it over palliative care. I would actually rather go out and shoot myself too.”* Four participants also misinterpreted palliative care as being reserved for those who can no longer look after themselves. P13 remarked, *“I’m pretty much a stick to myself sort of bloke. Independent. To me, none of that palliative care matters because I prefer to be by myself. I don’t think I’d like people working on me around the clock.”*

#### Palliative Care Stigma Sourced from the Experiences of Others

Participants often formed their perceptions of palliative care based on the personal experiences of people they knew. Eight respondents knew individuals in their communities who had negatively experienced palliative care, which hindered these participants from accepting palliative care due to its association with dying. For example, P6 commented, *“I’ve known of 3 people that have gone into palliative care that have not come out … So that’s the sort of thing just sitting at the back of my mind.”*

#### Cultural Misalignment with Palliative Care Principles

Cultural beliefs were occasionally identified as a barrier to palliative care uptake. Two out of ten participants of migrant background indicated palliative care conflicted with their belief systems. P2 revealed discussions pertaining to the end-of-life and death were cultural taboos. They remarked, *“In Chinese culture, totally against palliative care. Totally against talking about death.”* P7 believed the responsibility of healing should be reserved for God, stating, “*My faith in God is strong. Whatever God has written will come to pass. I don’t think about these things [death and palliative care]*.”

### Theme 2: Participants Lacked Awareness of Palliative Care

#### Limited Knowledge of Palliative Care

Participants’ knowledge of palliative care was often limited. Nine respondents reported having no knowledge of palliative care. These views were shared by both migrant and non-migrant participants (4/9 vs 5/9). Explaining palliative care for the first time to participants with limited knowledge of it led to the unanimous acceptance of palliative care (9/9).

#### Respondents Were Uncertain How to Access Specialist Palliative Care Services

Participants were uncertain how to access palliative care specialist services, with five respondents reporting they were unaware they could access it early in their disease course. Notably, eight respondents expressed interest in learning more about palliative care but had received little education from clinicians. For example, P18’s carer realised the value of palliative care managing her husband’s symptoms but was unsure how to access palliative care, questioning, *“At what stage can we access it? And how does it work?”*

### Theme 3: Participants Need Palliative Care Services

Participants frequently suffered from a multitude of issues that may have been mitigated by a comprehensive palliative care intervention, including symptom burden and poor care coordination. While some respondents rejected aspects of palliative care, all participants faced predicaments that could have been improved from at least one facet of palliative care.

#### Heavy Symptom Burden

All 21 participants struggled with the physical or psychological toll of hepatocellular carcinoma and its treatment. Commonly reported physical symptoms included severe abdominal and metastatic bone pain, ascites, nausea, and skin rashes (Table 3). Treatment side effects were also reported, with six participants complaining of headaches, weight gain and vomiting because of tyrosine kinase inhibitor medications. Respondents also reported feeling despondent due to their cancer diagnosis (n = 10). P17 described, “*It’s a mental battle … you want to get in life a bit more, but this [hepatocellular carcinoma diagnosis] just stops you because at the back of your mind, it’s the thought of, ‘well, you’re not gonna last anyway’*.” Eight respondents also shared the hindrance hepatocellular carcinoma has had on their social life and activities of daily living. For example, P11 described his susceptibility to infection from hepatocellular carcinoma treatment affecting his ability to see friends: *“I’ve got a bunch of mates … I haven’t been going out with them for a while. If somebody coughs in the back of the bus, I end up with something chronic.”*

#### Lack of care coordination

Six participants shared frustrations regarding the variable standard of care received at different care centres. These frustrations stemmed from uncertainty surrounding their prognosis and treatment plan, as clinicians commonly provided conflicting advice and information. For example, P1 suffered from primary lung cancer and hepatocellular carcinoma, and the respiratory and hepatology departments were involved in his care: *“I was anxious, only because I have lung cancer already. And then there was no communication between both departments … It took the lung department at least three weeks to get back to me. It was frustrating because you want to know what’s going on.”* Importantly, eight respondents identified uncertainty as a source of anxiety and stress.

#### Benefits Applicable to All

All 21 respondents reported having an aspect of their care that could have been addressed by palliative care, even if they rejected another facet of palliative care. For instance, three participants repudiated counselling. However, from this group, one individual had severely impacted activities of daily living, and the remaining two had experienced a lack of coordination of care and uncertainty surrounding their disease. P10 described the latter and realised the role palliative care could serve in this predicament: *“I can see the value of it [palliative care] now. They could have looked at my case as a whole, seen the fluid and then given us a way to drain it without going through emergency.”*

### Theme 4: Education Led to Acceptance of Palliative Care

Following an explanation of palliative care’s role in hepatocellular carcinoma (Table 2), the majority of respondents reported improved perceptions of palliative care (n = 17) (Table 4). This amelioration of attitudes arose from correcting and clarifying commonly held misconceptions, as described in Theme 1. However, four participants continued to spurn palliative care following education, rejecting the name ‘palliative care’.

**Table 4.** Changes in Participant Perception of Palliative Care Before and After Hearing a Standardised Definition of Palliative Care.

Participant Number	Illustrative Quote before Definition provided	Illustrative Quote after Definition provided
5	“End of the line. It’s like when you can’t look after yourself, you’re in palliative care someone will look after you to make sure you’re neat and tidy.”	“Yeah, you know what? I’d give palliative care a go to see what they had to offer.”
8	“Well to me it’s really people who can’t look after themselves and they need assistance pretty much 24 hours a day. And basically you’re in a palliative care environment where you’re not at home. End-of-life.”	“I didn’t realise it was sort of … what can I say. I Thought it was people further advanced in illness where they really couldn’t look after themselves. I didn’t know it could help healing.”
12	“It’s where you go when you die”	“That changes how I feel. Yeah, I’d accept it. It just sounds uplifting!”
16	“I think it’s people looking after you when you can’t look after yourself.”	“That’s a bit um … I thought it was just one main sort of thing, like only for people who need it, that sort of thing. It sounds a lot more holistic, you can have it any time.”
19	“Palliative care is when you’re in a situation where like … there’s an end. End-of-life.”	“Yeah, yeah that sounds good. I didn’t know it could help with all that, as you say.”
21	“I think for the palliative care, if a person has reached their end-of-life, they need more care, more support from other people.”	“Yeah, your definition’s changed how I felt about palliative care, in a good way”

Refer to Table 2 for standardised definition of Palliative Care.

#### Acceptance of Palliative Care Regardless of Ethnic or Religious Background

While two participants were initially opposed to palliative care due to cultural and religious beliefs, their reluctance was due to a misperceived notion of palliative care. However, after correcting these misconceptions with our provided definition, these participants accepted and altered their perceptions of palliative care. Importantly, following education, no participant cited cultural or religious beliefs as a reason to reject palliative care.

#### Palliative Care Education Should Commence at Diagnosis

Eleven participants preferred receiving education about palliative care as early as possible after diagnosis, arguing that this could serve to reframe palliative care in patients’ minds as holistic, quality-of-life focused care. P16 remarked, *“[Learn about it] as soon as you could. You’re the first person that’s told me about it. I understand the importance of it, how it can help with a lot of things, well now I do.”* Moreover, five participants opined that early access to all the information would have facilitated better health decisions. P19 commented, *“I suppose if you understood what palliative care was when you’re starting treatment, you’d kind of know at some stage that you might want to use it. It’s good to have already discussed it.”*

#### Renaming ‘Palliative Care’

Seven participants suggested renaming ‘palliative care’ to ‘supportive care’, to mitigate the stigma associated with the former. P16 commented, *“And the term itself … there’s a lot of terms we used to use before that we don’t use anymore. Because they’re sort of traumatic. I think palliative care is one of them.*” Of the four participants who rejected palliative care following the brief education, all four revealed that it was due to the name ‘palliative care’ and the discordance between our provided definition and their perception of it. P1 said, *“No, that doesn’t change how I feel. It’s still palliative care … you have to experience it before your perception changes … or you have to know someone that has experienced it like the way you say it.”*

## Discussion

### Main Findings

We conducted a qualitative study to delineate patient-perceived barriers to early palliative care integration in hepatocellular carcinoma. Participants discussed four key themes: (1) Palliative care is misperceived and stigmatised, (2) Participants lacked awareness of palliative care, (3) Participants need palliative care services, and (4) Education led to acceptance of palliative care.

### What This Study Adds

This study is the first to explore barriers to palliative care uptake among patients with hepatocellular carcinoma. Our description of patient-perceived stigma aligns with findings from studies conducted among other disease groups.^28,29^ Similar to our findings, patients in these studies sourced their palliative care misconceptions from the experiences of their community, indicating a pervasive public stigma surrounding palliative care. Significantly, palliative care continues to be perceived negatively as end-of-life care by the general public^31,32^ and medical profession^23,24,33^ alike. Stigma of any kind has been demonstrated to deleteriously impact health-seeking behaviour,^30^ as well as diagnostic, therapeutic and health outcomes.^34^ In this regard, patients are less likely to use palliative care services if they either experience palliative care stigma from clinicians or endorse it themselves.^35^

The second barrier identified was participants’ limited awareness of palliative care services, reflecting a prevalent theme in the literature with three studies demonstrating patients’ lack of understanding of palliative care processes.^28,29,36^ Significantly, previous studies have shown that palliative care intervention is often delayed in other diseases until patients request it.^21^ This notion, in combination with our results, suggests that patients with hepatocellular carcinoma might not be receiving timely palliative care, by virtue of their unawareness to request it.

Relatedly, patient stigma and unawareness of palliative care are barriers conceivably sourced from miseducation. Introducing a brief educational session elucidating palliative care and its role demonstrated a positive shift in palliative care perceptions. This mirrors previous research, where education was a powerful enabler of palliative care uptake by dispelling common misconceptions and stigma towards palliative care in end-stage liver disease and advanced cancer.^28,29^ Significantly, like our study, these studies explored perceptions of palliative care before and after a definition was provided, similarly demonstrating a temporal link between the educational intervention and amelioration of patient attitudes. Additionally, although our explanation of palliative care was brief, it resulted in participants expressing a desire for palliative care education to commence at diagnosis, a paradigm also seen in the referenced studies.^28,29^

Further, all participants who rejected palliative care following education revealed this was due to a discordance between our provided explanation and their persistent association of palliative care with end-of-life therapy. Consequently, participants supported renaming palliative care to bridge this dissonance. This deterrence to the name is evident in previous surveys, where participants preferred the name ‘supportive care’ over ‘palliative care’, regardless of the provided description.^37,38^ To highlight this, a palliative care unit in a United States hospital rebranded as ‘supportive care’, resulting in a significant 41% increase in referrals (*P* < 0.001).^39^ However, this may be difficult to realistically achieve, as palliative care is a globally recognised term with a well-established identity and history within the medical community^40^.

### Significance of Overall Findings

The findings of this study, supported by the results of previous qualitative studies,^28,29^ highlight the gap in knowledge of palliative care among patients with hepatocellular carcinoma and the effect a simple description of palliative care has on altering patient attitudes. In terms of improving palliative care uptake, a more accepting and informed patient population could guide and direct discussions surrounding palliative care referral. Existing literature indicates that clinicians delay initiating palliative care discussions until prompted by patients^21^ and face challenges in initiating such conversations due to a lack of resources or skills.^20,21,41^

This, combined with research that substantiates the benefits of integrating palliative care in the hepatocellular carcinoma treatment algorithm,^42^ underscores the need to develop a sound educational initiative to reframe palliative care as life-enhancing rather than life-ending therapy. This is necessary to improve the quality-of-life, survival, and disease outcomes of this traditionally underserved population. Furthermore, the education we delivered was brief, clarifying that a future interventions can be delivered with minimal time and resource strain. In fact, a US analysis of over 5000 patients demonstrated that palliative care involvement was associated with cost savings of USD$1696 per admission for patients who survived hospitalisation and USD$4098 per admission for patients who did not survive hospitalisation.^43^

Finally, renaming palliative care to ‘supportive care’ could improve uptake by alleviating stigma, a finding that has been reported in the literature.^37,38^ The association of palliative care with end-of-life care persists, despite the evolution of the specialty.^1,44^ However, more than 10 different definitions of “supportive care” exist, with wide variations in meaning. While a name change might bridge an initial reluctance to engage in palliative care services, it may not be realistic and would only likely lead to increased confusion^40^. It would also achieve little without a concurrent shift in how palliative care is understood.^40,45^ Therefore, renaming should be accompanied by an educational intervention to reframe public perception of palliative care.

### Limitations

Our study had limitations. Despite utilising purposive sampling to recruit a diverse sample, all respondents were recruited from a single health centre and so generalisability may be limited. The exclusion of individuals with hepatic encephalopathy also limits the generalisability of our data to this important subgroup of hepatocellular carcinoma patients, despite their potential to benefit significantly from early palliative care due to their severe symptom burden.^46^ Our final sample may have also skewed towards more favourable attitudes to palliative care, as three patients declined participation due to the palliative care content of the interviews, and two participants had previously received a palliative care consult.

### Future Directions

In light of these limitations, future research across several health centres utilising purposive sampling should be performed to improve generalisability. In addition, these studies should involve other key stakeholders, including caregivers and clinicians, whose perceptions may serve as barriers to the implementation of palliative care.

Considering this, while our study suggests that early palliative care education for patients with hepatocellular carcinoma could increase palliative care uptake, such education may not occur if physicians and caregivers are reluctant.^42^ Accordingly, physician training in palliative care principles is crucial, and future studies should explore how a patient education initiative could be best introduced and advocated for by clinicians. Moreover, caregiver perceptions in the hepatocellular carcinoma population are poorly defined, and understanding their convictions is vital for improving palliative care uptake, especially among those with decision-making roles. Therefore, future research is necessary to delineate caregiver beliefs and their perceived barriers to palliative care uptake.

## Conclusion

In a sample of 21 patients with hepatocellular carcinoma, we identified barriers to the uptake of early palliative care, with most participants reporting either limited knowledge or negative perceptions of palliative care. After a brief explanation, almost all participants accepted palliative care, and some supported a name change to mitigate stigma. Future studies are required to develop scalable interventions for education and a potential name change to overcome the widespread misperceptions of palliative care, and integrate it into hepatocellular carcinoma management.

## Supplemental Material

Supplemental Material - Exploring Misconceptions of Palliative Care Among Patients With Hepatocellular Carcinoma: A Pilot Study Supplemental Material for Exploring Misconceptions of Palliative Care Among Patients With Hepatocellular Carcinoma: A Pilot Study by Mostafa Abasseri, Shakira Hoque, Kim Caldwell, Linda Sheahan, Slavica Kochovska, Meera Agar, and Amany Zekry in American Journal of Hospice and Palliative Medicine®.

## Data Availability

Data sourced in authoring this manuscript is available within this article and is freely accessible via online medical databases.*
