# Supplementary material for: Exploring Misconceptions of Palliative Care Among Patients With Hepatocellular Carcinoma: A Pilot Study
Source: Am J Hosp Palliat Care. 2024 Aug 19;42(5):467–76. doi: 10.1177/10499091241268423 (PMC11894838; doi:10.1177/10499091241268423)
Supplement: Supplemental Material - Exploring Misconceptions of Palliative Care Among Patients With Hepatocellular Carcinoma: A Pilot Study [file sj-pdf-1-ajh-10.1177_10499091241268423.pdf]

Table 5. Coding Table and Illustrative Quotes derived from NVivo 12 (2018) thematic content analysis of interview data.

Themes	Subthemes	Line-by-line coding	Illustrative Quotes
Palliative care is misperceived and stigmatised	Participants believed palliative care is reserved for the end-of-life	Palliative care is reserved for the end-of-life	*"Palliative care is when you go in you don't come out. My mum went through it. They just pump you full of morphine till you don't work no more. It's basically euthanasia, slow euthanasia."*
		Prefer voluntary-assisted dying over Palliative care	*"I don't want to spend my last few days, weeks, whatever, in a hospital. I'm very pleased that the voluntary euthanasia thing is probably going to pass. I would rather it over palliative care. I would actually rather go out and shoot myself too." (P4)*
		Palliative care is when you cannot look after yourself	*"Palliative care basically is where you can't look after yourself, where you can't do normal day to day things like wash yourself, go to the toilet. That's my understanding of it." (P8)*
		Palliative care discussions should be reserved for end-of-life	*"We don't really want to think about that [palliative care] now. We want to be positive. Maybe later on." (P6)*
	Palliative care stigma sourced from the experiences of others	Experience of palliative care is different in various centres	*"But my experience, I used to visit people inside nursing homes, there's chalk and cheese – there's good ones and really bad ones. The really bad ones are the cheap ones, and I don't want to end up at one of these." (P11)*
		Work in aged care	*"I've seen in the aged care sector more so than hospitals, but in the aged care sector the way some of these poor buggers were treated. And the way that some of them are that shouldn't be there." (P6)*
		Friends who have entered palliative care do not come out	*"I've known of 3 people that have gone into palliative care that have not come out … Once they went in there, they weren't coming back out. So that's the sort of thing that's just sitting at the back of my mind." (P6)*
		People are usually already terminally ill when they enter palliative care which contributes to stigma	*"The people we know … they've only gotten it [palliative care] at the last bit of their life, and so maybe that makes people think it's for death." (P6)*

	Cultural misalignment with palliative care principles	Healing placed in the hands of God	*What is this [palliative care]? God is in control of all things. My faith in God is strong. Whatever God has written will come to pass. I leave everything to God. I don't think about these things [death and palliative care]." (P7)*
		Culture of silence	*"In Chinese culture, totally against palliative care. Totally against talking about death. So once you mention palliative care, we think 'I'm dying'. So we don't want it." (P2)*
Participants lacked awareness of palliative care	Limited knowledge of palliative care	No knowledge of palliative care	*I: "Is this the first time you're hearing of palliative care?"*<br>*P: "Yes, first time. I'm not clear on this palliative care." (P9)*
		Confused palliative care with something else	*I: "Do you know what it is?"*<br>*P: "Palliative care is going through that machine!" I: "Which machine?"*<br>*C: "The CT scan?" P: "Yeah."*<br>*C: "That's not palliative care." (P14)*
	Respondents were uncertain how to access specialist palliative care services	Unsure how to access early palliative care and how it works	*"But normally at what stage can we access it? And how does it work, does the patient have to live in the hospital or?" (P18)*
Participants need palliative care services	Heavy symptom burden	Heavy physical symptom burden of hepatocellular carcinoma and treatment	*"It's affected me a bit. My legs, the mets, the pain in the bone."(P7)*
		Anxiety and fear	*"One of the things about being diagnosed is everything becomes cancer. Lower back pain? Oh must be cancer. It's an anxious feeling." (P4)*
		Large impact on Activities of Daily Living	*"I can't exercise or work. I used to work 14 hours a week, now I do 4 hours." (P1)*
		Immunocompromised, no social life	*"I've got a bunch of mates, and we all used to ride Harleys … I haven't been going out with them for a while. It seems like if somebody coughs in the back of the bus, I end up with something chronic" (P11)*
		Concerns about family coping	*"My daughter she has been by my side forever. I'm worried about her. How will she cope if I die?" (P3)*

	Lack of care coordination	Uncertainty of prognosis and disease	*"My initial first day here, I saw a doctor, and I said to her "how long have I got", and she said "5 years". And I said well thank you very much. And I spoke to another doctor outside of the hospital and he said 'not true'. It's just conflicting. It's the uncertainty I guess." (P11)*
		Poor communication with healthcare providers	*"I was anxious. Only because I have lung cancer already. And then there was no communication between both departments … It took the lung department at least 3 weeks to get back to me. It was frustrating – because you want to know what's going on." (P1)*
		Unhappy with care	*"It's been bad. Very average. I was sat in bed, no blanket, freezing cold. I'd been up all night stuck in a chair on a corridor. Then 3 doctors come and say 'you've got cancer'. Like that's that. She said we want to do a biopsy but we have to wait till over the weekend. I just left." (P6)*
	Benefits applicable to all	Rejects counselling but accepts care coordination	*"No. Don't want counselling. Wrong attitude for it." (P10)*<br><br>*"I can see the value of it [palliative care] now. They could have looked at my case as a whole and seen the fluid [ascites] and then given us a way to drain it and manage it without having to go through emergency and the extra referrals." (P10)*
		Rejects pain medication but accepts counselling	*"All I've known is pills, pills, pills. I've been heavily on endone, panadeine forte, Valium and Lyrica for nerve pain, anti-inflammatories for the arthritis. Since all that pain left, I was like no more. I told them [hepatologists], you're not feeding me these tablets anymore." (P5)*<br><br>*"Shock was my first feeling, anxious … The stress will kill you … I have a counsellor." (P5)*

		Rejects counselling but impacted activities of daily living	*"No. waste of time. It'd be talking to that wall. You know what I mean? I don't care what they believe it's none of my business, that's why I never stick my nose in other people's business. Some people need these people. A lot of people that are sick. No more spiritual stuff." (P10)*<br>*"This cancer … I can't do swimming anymore. I can't walk." (P10)*

Education led to acceptance of palliative care	Acceptance of palliative care regardless of ethnic or religious background*	Regardless of ethnic or religious background, participants accepted palliative care following education	*"I didn't realise it was sort of … what can I say. I thought it was people further advanced in illness where they really couldn't look after themselves."* – Caucasian Christian male (P8)<br>*"That changes how I feel. Yeah it does. Because, I don't know. It just sounds uplifting!"* – Caucasian nonreligious male (P12)<br>*"yes, your definition changed how I felt about palliative care in a good way."* – Chinese Buddhist male (P21)<br>*"I think it is a good thing."* – Chinese nonreligious male (P20)<br>*"Yeah, it's okay. I like that."* – Bengali Muslim male (P9)
	Palliative care education should commence at diagnosis	Mitigates uncertainty	*"I think right at the start. You need to give people the option, like what are the option? If the treatment doesn't work, then that's the option of natural course. It would help with the uncertainty, you know, knowing all the facts." (P1)*
		Prevention is better than cure	*"When would be the best time for anybody to learn about anything? Yesterday. Not when it happens. Prevention is better than cure! Don't wait till it happens." (P5)*
		To make better decisions for their health	*"I think that people should learn about it very early to increase their knowledge. So they can make better decisions." (P20)*
	Renaming 'Palliative Care'	Renaming palliative care would make participants more comfortable	*"Yes. If you avoid the sensitive word 'palliative care', we would feel much better." (P2)*
		The term palliative care is confronting	*"And the term itself … there's a lot of terms we used to use before that we don't use anymore. Because they're sort of… traumatic. I think palliative care is one of them." (P16)*
		Did not know what 'palliative meant'	*"I: When you hear the term palliative care, what's the first thing that comes to your mind?*<br>*P: Uhm, they stick you in a palate and throw you in a grave. I don't know what palliative means, I only make jokes about being on a palate. It seems like you're on your way to [name of palliative care hospital]." (P12)*

I, Interviewer; P, Participant; C, Carer. *Quotes of individuals from various ethnic backgrounds accepting palliative care following provision of definition were included to illustrate this theme. Note, this coding table is not exhaustive and does not include all illustrative quotes from interviews.
